# Human D-Amino Acid Oxidase: Structure, Function, and Regulation

**DOI:** 10.3389/fmolb.2018.00107

**Published:** 2018-11-28

**Authors:** Loredano Pollegioni, Silvia Sacchi, Giulia Murtas

**Affiliations:** Dipartimento di Biotecnologie e Scienze della Vita, Università degli Studi dell'Insubria, Varese, Italy

**Keywords:** D-amino acid oxidase, D-serine, substrate specificity, structure-function relationships, NMDA receptor

## Abstract

D-Amino acid oxidase (DAAO) is an FAD-containing flavoenzyme that catalyzes with absolute stereoselectivity the oxidative deamination of all natural D-amino acids, the only exception being the acidic ones. This flavoenzyme plays different roles during evolution and in different tissues in humans. Its three-dimensional structure is well conserved during evolution: minute changes are responsible for the functional differences between enzymes from microorganism sources and those from humans. In recent years several investigations focused on human DAAO, mainly because of its role in degrading the neuromodulator D-serine in the central nervous system. D-Serine is the main coagonist of N-methyl D-aspartate receptors, i.e., excitatory amino acid receptors critically involved in main brain functions and pathologic conditions. Human DAAO possesses a weak interaction with the FAD cofactor; thus, *in vivo* it should be largely present in the inactive, apoprotein form. Binding of active-site ligands and the substrate stabilizes flavin binding, thus pushing the acquisition of catalytic competence. Interestingly, the kinetic efficiency of the enzyme on D-serine is very low. Human DAAO interacts with various proteins, in this way modulating its activity, targeting, and cell stability. The known properties of human DAAO suggest that its activity must be finely tuned to fulfill a main physiological function such as the control of D-serine levels in the brain. At present, studies are focusing on the epigenetic modulation of human DAAO expression and the role of post-translational modifications on its main biochemical properties at the cellular level.

## Introduction

**Figure 1:**
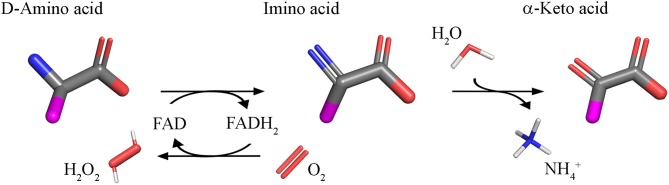
Reaction catalyzed by DAAO.

Using FAD as cofactor, D-amino acid oxidase (DAAO, EC 1.4.3.3) catalyzes with strict stereoselectivity the oxidative deamination of neutral D-amino acids. DAAO has been discovered in pig kidney in 1935 (Krebs, 1935) and during the years it has been investigated as a prototype of FAD-dependent oxidases and has been the object of a plethora of studies: 96,325 publications concerning DAAO have appeared over the years (Scopus, 1 October 2018) with a significant increase from 2000 onward. D-Amino acids are dehydrogenated by DAAO into imino acids that spontaneously hydrolyzed to the corresponding α-keto acids and ammonia; the reoxidation of FADH_2_ on molecular oxygen generated hydrogen peroxide (Figure 1).

The reaction catalyzed by DAAO is of biotechnological relevance since it can be used in biocatalysis (to produce α-keto acids from D-amino acids or 7-aminocephalosporanic acid from cephalosporin C, to resolve racemic mixtures of natural and synthetic amino acids, etc.), in biosensors, and in cancer therapy, to mention only the main applications (Pilone and Pollegioni, 2002; Caligiuri et al., 2006a,b; Pollegioni and Molla, 2011). For such a use, DAAO was isolated from microorganisms: those from *Trigonopsis variabilis* and *Rhodotorula gracilis* have been investigated in depth (Pollegioni et al., 2002, 2008; Arroyo et al., 2007).

The investigations on DAAO from higher organisms started in the 1980s. The enzyme's physiological role was long debated largely because the levels of D-amino acids were barely detectable and their presence in many tissues was questioned. Later on, appreciable levels of various D-amino acids were determined in brain and other tissues based on improved analytical methods (mainly high-performance liquid chromatography) (Nagata, 1992; Nagata et al., 1992; Hashimoto et al., 1993; Hamase et al., 1997). This cleared the path to identifying specific physiological roles for D-amino acids (Wang et al., 2000; Wolosker et al., 2002; Fuchs et al., 2005) and to propose for DAAO a key role in their metabolic control. Here, the ability of D-serine to act in the central nervous system as a coagonist of N-methyl D-aspartate receptors (NMDAR), excitatory amino acid receptors critically involved in learning and memory, stimulated the field.

D-Serine is mainly synthesized in neurons by racemization of the L-enantiomer catalyzed by the pyridoxal phosphate-dependent enzyme serine racemase (SR, EC 5.1.1.18) (Wolosker et al., 1999). L-Serine is provided by astrocytes possessing a specific metabolic pathway, referred to as the “phosphorylated pathway,” the primary route for the net synthesis of L-serine in the brain, considering the low permeability of the amino acid at the blood-brain barrier (Furuya et al., 2000). SR can also catabolize D- and L-serine through an α,β-elimination reaction to give pyruvate (Foltyn et al., 2005). From a cellular point of view, SR is a “complex” enzyme since its activity is modulated by energy level (ATP), metal ions, post-translational modifications, and protein interactors; for details, see (Pollegioni and Sacchi, 2010; Conti et al., 2011; Dellafiora et al., 2015; Beato et al., 2016). Once released by neurons, D-serine is rapidly taken up and stored in astrocytes (Wolosker, 2011; Wolosker and Radzishevsky, 2013). SR is poorly expressed in astrocytes, which instead produce DAAO; in these cells the flavoenzyme indirectly controls its availability at the synapse by regulating D-serine cellular concentrations and affects the activation level of NMDAR by modulating the occupancy of the co-agonist site.

## Role of DAAO in physiological and pathological conditions

*DAO* gene is present in a single copy in human chromosome 12 (12q23-24 region) (Konno, 2001, AA): its structure has been detailed in Figure 3 of Pollegioni et al. (2007). A definite report of the *DAO* gene and protein expression in human tissues, with a particular focus on the brain regions, was recently reported, see Table 1 in Sacchi et al. (2018) and Figure 2 of Molla (2017). The highest amount of DAAO protein is observed in liver and kidney: in the latter organ, the enzyme is expressed in proximal tubule cells (Koibuchi et al., 1995; Sasabe et al., 2014a). DAAO was associated with chronic, pathologic renal damage, e.g., D-serine and D-propargylglycine induced nephrotoxicity due to DAAO-mediated generation of H_2_O_2_ (Konno et al., 2000; Maekawa et al., 2005; Krug et al., 2007).

The elucidation of the physiological functions of DAAO was accelerated by investigating the mutant ddY/DAAO^−/−^ mice strain expressing the inactive G181R enzyme variant (Konno and Yasumura, 1983): large amounts of D-amino acids were excreted in the urine of these animals. Accordingly, in liver and kidney (as well as in the urinary tract and in colon) DAAO eliminates D-amino acids originating in the cell walls of intestinal bacteria, from endogenous racemization, or from the diet. Indeed, increased D-serine levels were apparent in brain regions normally characterized by high DAAO expression in wild-type animals (Morikawa et al., 2001; Miyoshi et al., 2009).

In kidney and brain, the flavoenzyme is a component of the DAAO/3-MST pathway related to hydrogen sulfide (H_2_S) generation (Shibuya et al., 2013). Within peroxisomes, DAAO metabolizes D-cysteine (mostly provided by food) to 3-mercaptopyruvate, which is then imported into mitochondria where it is converted to H_2_S by 3-mercaptopyruvate sulfurtransferase (3MST). H_2_S regulates kidney excretory function and modulates blood pressure by affecting the release of renin.

The expression of DAAO was also reported in the granule fraction of mature human granulocytes (specifically on the cell surface), where it was proposed to participate in recognizing and counteracting foreign, phagocytosed microorganisms (Cline and Lehrer, 1969; Robinson et al., 1978). Within the phagosome, DAAO metabolizes D-alanine (derived from the peptidoglycan of the bacterial cell wall) producing H_2_O_2_, which in turn is the oxidant substrate for myeloperoxidase that converts chloride to hypochlorous acid, a strongly microbicidal compound. Compared to the wild-type strain, the aforementioned ddY/DAAO^−/−^ mice show a higher susceptibility to *S. aureus* infection (Nakamura et al., 2012).

It was recently reported that DAAO plays a role in controlling the homeostasis of gut microbiota (Sasabe et al., 2016): DAAO (protein and activity) was identified in the proximal and middle small intestine of mice and humans, associated to the villus epithelium. A proteolyzed form of mouse DAAO was reported to be secreted in the lumen by goblet cells: this extracellular form is likely secreted by an N-terminal signal peptide and cleaved at the level of a putative cleavage site also located at the N-terminus (Sasabe et al., 2016). The H_2_O_2_ generated by DAAO during the catabolism of free D-amino acids of microbial origin represents an important factor in host defense (it protects the mucosal surface from the cholera pathogen) and in modifying microbiota composition (Sasabe et al., 2016).

In the central nervous system, DAAO is the enzyme mainly responsible for catabolism of D-serine: notably, in rodents and humans DAAO expression mirrors D-serine distribution. DAAO (activity and immunoreactivity) was mainly detected in cerebellum and, at lower levels, in the forebrain (Verrall et al., 2007; Madeira et al., 2008). A quite recent investigation confirmed DAAO expression in human forebrain regions and, at the same time, also highlighted that its activity is present in the white matter, throughout the corticospinal tract, and in the spinal gray matter, where it is localized in astrocytes mainly situated in the motor pathway (Sasabe et al., 2014b). The significant hDAAO activity assayed in spinal cord and brain stem is coherent with the proposed function in preventing excitotoxic cell death.

Morover, hDAAO activity was identified in dopaminergic neurons of the nigrostriatal system (Sasabe et al., 2014b): hDAAO is known to efficiently metabolize D-DOPA (see below); thus, the enzyme could affect the metabolism of dopamine, norepinephrine, and epinephrine.

In spinal cord neurons, NMDARs are expressed and are involved in the development of ongoing pain states via central sensitization (Latremoliere and Woolf, 2009). The tonic, pain-related behavior was amplified in the ddY/DAAO^−/−^ mice strain (Wake et al., 2001): the boost in the second phase of the formalin response is due to the potentiated NMDAR activation by the ensuing increased D-serine concentration. Later on, the role of DAAO as a pronociceptive factor in the spinal cord was confirmed (Zhao et al., 2010; Gong et al., 2011). Notably, the administration of DAAO inhibitors in rat models of tonic and chronic pain reversed pain-related behaviors and decreased the electrophysiological activity in spinal cord dorsal horn neurons and peripheral afferent inputs (Hopkins et al., 2013a). Among the putative ways in which DAAO is involved in chronic pain, a change in local levels of reactive oxygen species has been reported for formalin-induced pain (Lu et al., 2012). In this case, by inhibiting DAAO activity, a decrease in the production of spinal H_2_O_2_ levels is apparent (Lu et al., 2012; Gong et al., 2014). Interestingly, spinal DAAO has been also involved in pain hypersensitivity induced by perturbing sleep-regulating circuitries in the central nervous system through the deprivation of sleep, a process that generates pain hypersensitivity with no nerve or tissue injury (Wei et al., 2013). The H_2_O_2_ generated by DAAO could target the pronociceptive TRPA1 channel expressed by central terminals of primary afferent nerve fibers in the spinal dorsal horn.

Amyotrophic lateral sclerosis (ALS) is a rapidly progressing, adult-onset, neuromuscular disease distinguished by the selective loss of motor neurons. A recent, comprehensive, exome-sequencing study revealed that the only DNA variants associated with clinical outcome of ALS and with lower rates of survival are located on the *DAO* gene (Cirulli et al., 2015). Actually, the R199W DAAO substitution was identified in a three-generational familial ALS (fALS) kindred (Mitchell et al., 2010). This substitution impaired DAAO activity, boosted the formation of ubiquitinated protein aggregates, promoted autophagy activation, and increased apoptosis when the protein was overexpressed in motor neuron cell lines or primary motor neuron cultures (Paul and de Belleroche, 2012; Paul et al., 2014). The transgenic mouse lines expressing R199W DAAO (DAO^R199W^) were unaffected in survival although they exhibited the features common to several ALS mice models, i.e., decreased body weight, marked kyphosis, and loss of motor neurons in spinal cord (Kondori et al., 2017). Recently, it was reported that the most significant and robust splicing change after depletion of hnRNP A2/B1 in the mouse spinal cord was the skipping of exon nine within *DAO* gene, yielding a reading frameshift and early termination of the protein, predicted to lack 2 α-helices and 3 β-sheets and to generate a highly unstable and inactive variant (Martinez et al., 2016).

Impaired NMDAR signaling pathways are known to occur in the hippocampus and cerebral cortex of aging brains (Billard, 2008); in aged tissues a hypoactivation of NMDAR is related to decreased D-serine levels (Junjaud et al., 2006; Mothet et al., 2006). Neurodegeneration induced by NMDAR hypoactivity was also proposed to contribute to AD and to be related to the progression of aging brain from mild cognitive impairment to AD (Olney et al., 1997; Wozniak et al., 1998). Compared to healthy individuals, the serum levels of DAAO are increased in patients affected by mild cognitive impairment and mild and severe AD (Lin et al., 2017), and DAAO levels correlate with the severity of cognitive deficit and with the D-serine level.

Alterations in D-serine levels have been observed in Alzheimer's disease (AD) and have been suggested to represent a pro-death signal (Billard, 2008; Madeira et al., 2015).

An NMDAR hypofunction was also related to schizophrenia (Coyle et al., 2003; Coyle, 2006; Stone and Pilowsky, 2007): the altered activation state of the receptor was proposed to depend on a deficiency in D-serine signaling (Hashimoto et al., 2003, 2005; Verrall et al., 2010). The protein and activity levels of hDAAO were altered in post-mortem brain tissues from schizophrenic patients in cerebral cortex (Madeira et al., 2008), cerebellum (Kapoor et al., 2006; Verrall et al., 2007; Burnet et al., 2008), medulla oblongata, and choroid plexus (Ono et al., 2009). Further support comes from the discovery that the *G72* gene, encoding the small protein pLG72, the main hDAAO-specific binding protein (see below), has been linked to schizophrenia (Chumakov et al., 2002; Sacchi et al., 2008, 2016; Pollegioni et al., 2018). Additional meta-analyses supported a genetic association between *DAO, G72*, and schizophrenia: they have been classified as schizophrenia susceptibility genes (Sacchi et al., 2016).

## Cellular properties of hDAAO

DAAO is considered a marker of peroxisomes since it contains a classical PTS1 signal at the C-terminus (Horiike et al., 1994; Moreno et al., 1999). Notably, an active DAAO form has been reported in the cytosol, both in glial cells and neurons (Sacchi et al., 2008, 2011; Popiolek et al., 2011). In astrocytes overexpressing hDAAO, the cytosolic form seems to transiently accumulate in this compartment before targeting peroxisomes (Sacchi et al., 2011). Recent reports on rats demonstrated that DAAO is present both in the cytosol and nuclei of proximal tubule epithelial cells following treatment with the drug propiverine (Luks et al., 2017a,b) and that intestinal epithelial cells in mice secrete the flavoenzyme into the lumen (Sasabe et al., 2016).

The degradation pathway of hDAAO was investigated in U87 glioblastoma cells stably expressing the flavoenzyme fused to the C-terminus of the enhanced yellow fluorescent protein (EYFP, thus generating a peroxisomal chimeric protein) or at the N-terminus (thus producing a cytosolic chimeric protein since the PTS1 signal is masked). hDAAO is a long-lived protein showing a half-life > 60 h. The peroxisomal EYFP-hDAAO is degraded via the lysosomal/endosomal pathway, whereas the cytosolic hDAAO-EYFP protein is ubiquitinated and targets the proteasome. Overexpression of the interacting protein pLG72 (showing a rapid turnover, half-life in the 25–40 min range) increases the turnover of DAAO (half-life ~6 h) (Sacchi et al., 2011): hDAAO-pLG72 complex formation seems to represent a means to play a protective role against excessive D-serine depletion by the active, cytosolic enzyme (see below).

## Biochemical properties

### General properties

A comparison of the main biochemical properties of mammalian DAAOs is reported in Sacchi et al. (2012). Recombinant hDAAO is produced in fairly large amounts in *E. coli* cells (Kawazoe et al., 2006; Molla et al., 2006; Romano et al., 2009). It is purified as active holoenzyme by adding exogenous FAD to the purification buffers: hDAAO shows the classical properties of flavoprotein oxidases, such as a quick reaction with O_2_ in the reduced form and stabilization of the anionic red flavin semiquinone.

In the 6–10 pH range, hDAAO shows a good activity and stability (Murtas et al., 2017b). From the fitting of the activity values determined at different pH values, two dissociations were apparent: a pK_a_ of 2.5 and 11.1, respectively. Notably, the enzyme is fully stable after 60 min of incubation at 4°C at pH values ≥3.0 and ≤ 10.0. The flavoenzyme is stable up to 45°C, a temperature corresponding to the optimum for the enzymatic activity. The melting temperature determined following the loss of activity was ~55°C (Murtas et al., 2017b), higher than the values determined using the changes in protein fluorescence intensity (Caldinelli et al., 2010): this result suggests that the alteration in protein conformation brings forward the loss of enzymatic activity.

hDAAO activity is not affected by the presence of divalent ions (Ca^2+^ and Mg^2+^) and/or nucleotides. Similarly, the reducing agent N-acetyl-cysteine, a derivative of L-cysteine, acting as antioxidant and anti-inflammatory agent and that is able to modulate NMDAR activity (Kumar, 2015), does not affect the activity of hDAAO (Murtas et al., 2017b).

### Substrate specificity

**Figure 2:**
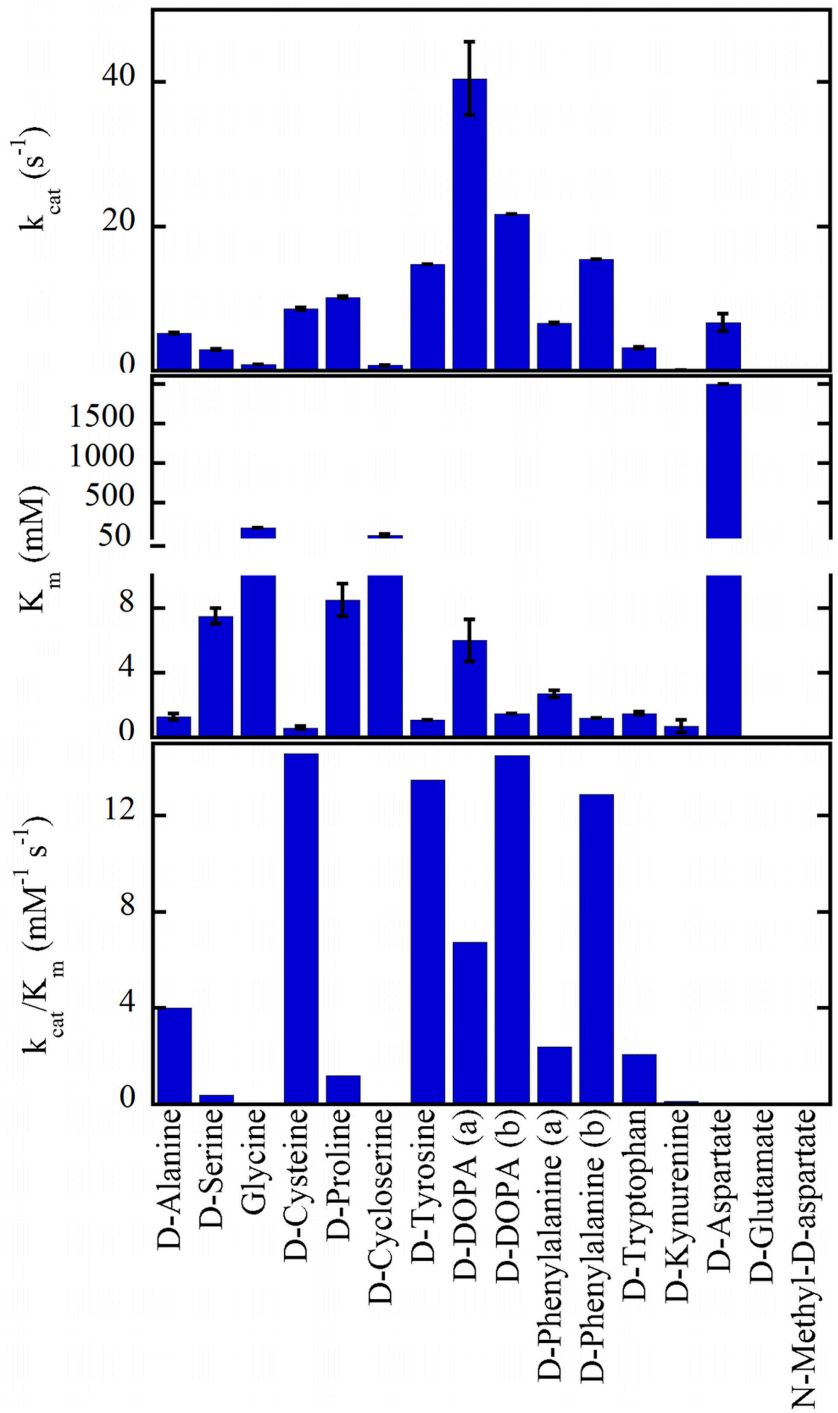
Substrate preference of hDAAO. The apparent kinetic properties have been determined at 21% oxygen saturation, pH 8.5, and 25°C. All values were from Molla et al. (2006) with the exception of (a) Murtas et al. (2017b), Frattini et al. (2011); (b) Kawazoe et al. (2007b).

hDAAO shows a wide substrate acceptance: the best substrates are hydrophobic and bulky D-amino acids (D-DOPA > D-Tyr > D-Phe > D-Trp, Figure 2). The highest *k*_*cat*_ value was determined for D-3,4-dihydroxy-phenylalanine (D-DOPA) (Kawazoe et al., 2007a,b; Murtas et al., 2017a,b), also showing a high affinity due to two additional H-bonds between the OH-groups of the substrate and His217 and Gln53 (Kawazoe et al., 2007a). However, the oxidation of D-DOPA is hindered by the substrate inhibition effect, K_i_ of 0.5 (Murtas et al., 2017b) or 41mM (Kawazoe et al., 2007b).

hDAAO is also active on small, uncharged D-amino acids (D-Cys > D-Ala > D-Pro > D-Ser) (Molla et al., 2006; Kawazoe et al., 2007b; Frattini et al., 2011; Murtas et al., 2017b). Purified recombinant hDAAO shows a low catalytic efficiency on what is known as the main physiological substrate, D-serine. Whether *in vivo* (and especially in glial cells) an increase in kinetic efficiency is achieved by the binding with a cellular compound (i.e., a protein or a small size ligand) or by a post-translational modification is still unknown: this issue deserves further investigations. The highest catalytic efficiency was determined for D-cysteine, a compound involved in H_2_S generation (see above) (Shibuya et al., 2013).

hDAAO is not active on glycine and acidic D-amino acids (NMDA and D-Glu) while the activity on D-Asp is hampered by the high apparent K_m_ (in the molar range) (Molla et al., 2006; Murtas et al., 2017b). hDAAO also oxidizes D-kynurenine with an apparent K_m_ value (0.7mM) resembling that determined for D-cysteine. Kynurenic acid, the product of D-kynurenine oxidation, binds to the modulatory glycine site of the NMDAR resulting in an inhibitory effect. Furthermore, hDAAO is also active on D-cycloserine, an NMDAR modulator (Kumar, 2015).

The substrate promiscuity of hDAAO supports the hypothesis that this flavoenzyme might play a role in different tissues and cells.

The activity of hDAAO on D-serine is partially inhibited by the L-enantiomer (Murtas et al., 2017b). L-Serine acts as competitive inhibitor (K_i_ of 26.2 mM). Under anaerobic conditions L-serine, as well as L-alanine or L-valine, are able to reduce FAD. However, a physiological concentration of L-serine (corresponding to ≤2 mM in brain tissues and in blood) (Weatherly et al., 2017) should not modify the oxidation of D-serine by hDAAO.

### Kinetic mechanism

**Figure 3:**
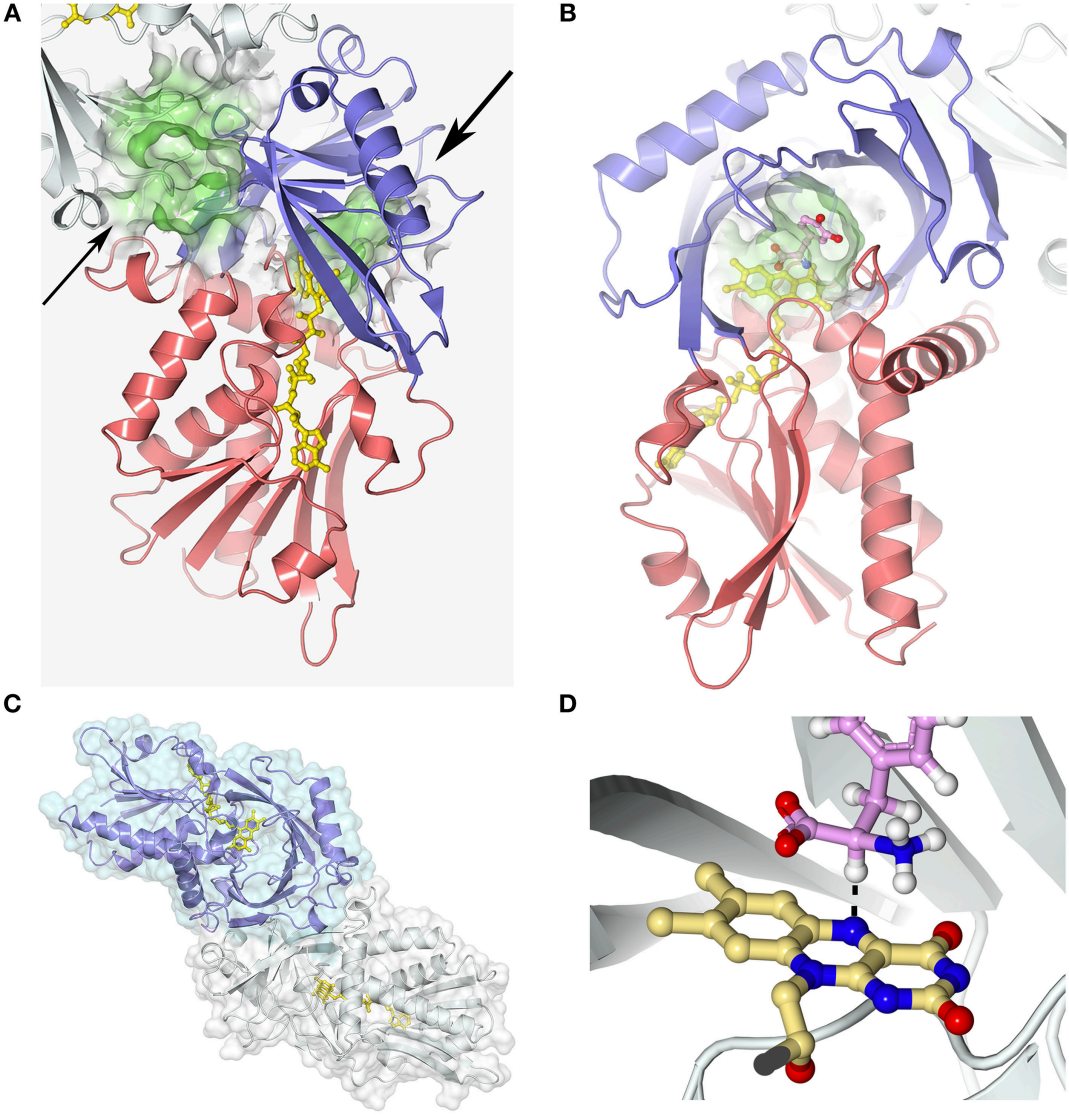
hDAAO three-dimensional structure (pdb codes 2e49). **(A)** The hDAAO protomer is constituted by two domains: the substrate and the FAD-binding domain (in blue and red, respectively). The entrance to the active site is indicated by a large arrow. The thin arrow indicates the putative additional ligand-binding site (Kohiki et al., 2017). **(B)** The substrate is located above the *re*-side of the isoalloxazine ring of FAD, in a cavity of ~220 Å^3^. **(C)** hDAAO is a stable homodimer, characterized by a head-to-head mode of monomer interaction (Kawazoe et al., 2006). **(D)** The substrate dehydrogenation ensues by the direct hydride transfer of the α-H from the α-C of the D-amino acid to the flavin N(5), see dotted line (Umhau et al., 2000). Following hydride transfer, a negative charge is generated on the reduced flavin, which is stabilized by the positive charge generated on the imino group of the product. This figure has been generated by modeling a D-Tyr molecule instead of the original ligand imino serine. Figure prepared with 3dproteinimaging.com.

For all known DAAOs, the oxidative deamination of D-amino acids follows a ternary-complex mechanism (Pollegioni et al., 1993; Umhau et al., 2000; Molla et al., 2006). The substrate dehydrogenation ensues by the direct hydride transfer of the α-H from the α-C of the D-amino acid to the flavin N(5): please see below and Figure 3D. The distance between these atoms is 3.6 Å in the hDAAO-imino serine complex: owing to the tetrahedral geometry of the substrate α-C, the mentioned atoms should be closer in the Michaelis complex (~3.2 Å). Following hydride transfer, the reduced flavin is negatively charged: the positive charge of the product imino group electrostatically stabilizes the reduced cofactor.

For mammalian DAAOs, and especially for hDAAO, the first half of the reaction (the reductive half-reaction), namely, the conversion of the tetrahedral D-amino acid into the planar imino acid coupled to the flavin reduction is fast (117 ± 6 s^−1^ on D-serine), significantly faster than turnover (6.3 ± 1.4 s^−1^). The rate-limiting step in hDAAO catalysis is the product release (Molla et al., 2006; Molla, 2017). The rate of imino acid release from the reduced enzyme is < 1 s^−1^, too slow to allow the reoxidation step to start from the free, reduced enzyme. Accordingly, reoxidation must start from the corresponding reduced enzyme-imino acid complex: the second-order reaction corresponds to 1.25 × 10^5^ M^−1^s^−1^.

## HDAAO structural-functional properties

### Overall structure

Each hDAAO protomer contains 347 amino acids (40.3 kDa), harbors one molecule of FAD, and is composed of 11 α-helices and 14 β-strands. hDAAO is constituted by two interconnected regions: an FAD-binding domain containing the dinucleotide binding motif (Rossman fold) and a substrate-binding domain in which a large, twisted, antiparallel β-sheet forms the active-site roof and part of the oligomerization interface (Figures 3A,B). hDAAO is a stable homodimer: the two monomers interact via a head-to-head geometry (Figure 3C; Kawazoe et al., 2006).

In the active site, the substrate is located above the *re*-side of the isoalloxazine ring of FAD, in a cavity of ~220 Å^3^ (Figure 3B). The substrate is bound via several hydrogen bonds in the correct orientation with respect to the flavin N(5) position for catalysis to proceed: the α-carboxylic group of the substrate electrostatically interacts with Arg283 and Tyr228, whereas the α-amino group interacts with Gly313 and the C(4) = O of FAD. The side chain of the substrate is placed in a pocket made up of hydrophobic residues (Leu51, Gln53, Leu215, and Ile230), named the substrate-specificity pocket (Kawazoe et al., 2006). The active-site “roof” is shaped by the side chain of Tyr224, a residue belonging to a mobile loop (216–228): the product/substrate exchange during catalysis is facilitated by the switch of this residue from a closed to an open conformation. This conformational change significantly influences the enzyme properties: limiting the turnover, increasing the hydrophobicity of the active site, and allowing hDAAO to bind large substrates (Molla et al., 2006; Kawazoe et al., 2007b).

The strict stereoselectivity of DAAO for the D-enantiomer of the amino acids is rationalized by the four-location model for enantioselectivity (Mesecar et al., 2000; Umhau et al., 2000; Mörtl et al., 2004). According to this model, the substrate establishes three interactions—using the α-carboxylic group, the α-amino group and the side chain—with the active site residues indicated above: the exact binding produces a “functional direction” exemplified by the alignment of the α-H of the substrate and the N(5) of FAD, which allows hydride transfer (Figure 3D).

### Oligomeric structure

Different from other DAAOs (Mattevi et al., 1996; Pollegioni et al., 2007; Frattini et al., 2011), an 80 kDa homodimer is generated by both the holo- and the apoprotein form of hDAAO (Molla et al., 2006). This results from a distinguishing charge distribution at the dimer interface (a region corresponding to ~1,500 Å^2^, i.e., the 9.8% of the overall solvent accessible surface, Figure 3C), where a significantly higher amino acidic substitution frequency was observed than for the overall protein (33 vs. 15%, respectively) (Kawazoe et al., 2006). Notably, the urea-induced dissociation of dimeric hDAAO generates protein conformers prone to aggregation (Caldinelli et al., 2009).

### FAD binding

In hDAAO the FAD cofactor shows an elongated conformation and it is buried in the protein core: the isoalloxazine ring is located at the interface between the two subdomains with the *re*-side facing the interior of the active site (Kawazoe et al., 2006; Molla, 2017). At this side of the flavin ring, the conformation of the surrounding residues is highly conserved among mammalian DAAOs. Conversely, at the *si*-face, the conformation of the hydrophobic stretch (47-VAAGL-51, a structurally ambivalent peptide) differs between the human and porcine enzymes, causing loss of the H-bond between Ala49 and N(5) of the cofactor and likely decreasing the strength of the interaction of the flavin cofactor and the rate of flavin reduction (Kawazoe et al., 2006).

hDAAO possesses the weakest binding of the FAD cofactor (K_d_ = 8.0μM) among known DAAOs (K_d_ = 0.2 and 0.02μM for pig and yeast DAAOs, respectively). Accordingly, hDAAO exists in solution as an equilibrium of holo- and apoprotein forms (Caldinelli et al., 2010). The presence of an active-site ligand increases the affinity of the flavin to the protein moiety, K_d_ = 0.3μM (Molla et al., 2006; Caldinelli et al., 2010). Quenching of protein fluorescence intensity during titration of the apoprotein with the cofactor, in the presence or absence of sodium benzoate, is a biphasic process (Murtas et al., 2017b), suggesting that the apoprotein form exists in two conformations with differing cofactor binding affinity: the higher intensity amplitude associated with the first phase observed in the presence of benzoate indicates that binding of an active-site ligand favors the protein conformation with the higher avidity for FAD (Murtas et al., 2017b). A second possibility is the presence of two binding sites. Here, a recent investigation based on computational and labeling analyses suggests that an additional ligand-binding site is located at the monomer-monomer interface (Figure 3A; Kohiki et al., 2017).

The holoenzyme reconstitution is a sequential process: in the first step, FAD binds the apoprotein moiety and recovers the catalytic activity; in the second step, a slow, secondary conformational change generates the final holoenzyme conformation (Caldinelli et al., 2009). Notably, the first step is 20-fold faster when benzoate is present (Caldinelli et al., 2010).

The melting temperature for the unfolding of the holoenzyme is 6–9°C higher than for the corresponding apoprotein (Caldinelli et al., 2009).

The observed increase in cofactor binding affinity in the presence of benzoate suggested that, in addition to the different conformation of the hydrophobic VAAGL sequence observed in the hDAAO-benzoate complex (Kawazoe et al., 2006), an alternative conformation of the substrate-free enzyme should exist that binds the cofactor less efficiently (Murtas et al., 2017b). In any case, the structure of the free enzyme form (PDB 2e48) overlaps that of the hDAAO-benzoate complex (PDB 2du8).

Based on the *in vivo* concentration of FAD (~5 μM), it is conceivable that in the cell an equilibrium between the hDAAO holoenzyme (active) and the apoprotein (inactive) form exists, with the latter one being predominant in the absence of an active-site ligand.

### Ligand binding

**Figure 4:**
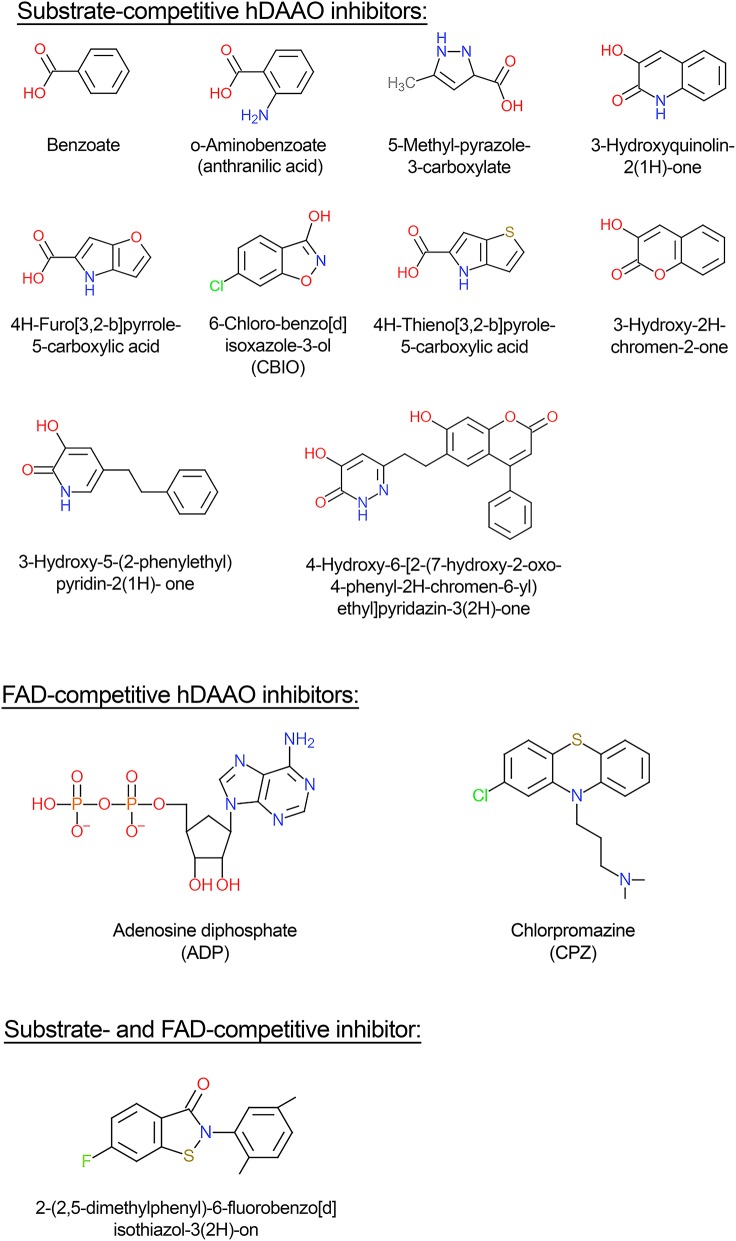
Structural formula of selected hDAAO inhibitors classified based on their mechanism of enzyme inhibition.

hDAAO inhibitors can essentially be grouped into substrate-competitive and cofactor-competitive inhibitors (Molla et al., 2006; Sacchi et al., 2012; Terry-Lorenzo et al., 2014; Molla, 2017). Among the active-site ligands, small aromatic (aryl) carboxylic acids or acid isosteres are powerful hDAAO inhibitors used as scaffolds for developing novel drugs (see below). These compounds, such as benzoate, *o*-aminobenzoate, substituted quinilinones, or 4H-furo[3,2-*b*]pyrrole-5-carboxylic acid (Figure 4), bind the flavoenzyme similarly to the substrate: the inhibitor COOH group (or the corresponding C = O or OH substituents) interacts with Arg283 and Tyr228, an H-bond donor binds Gly313, and the remaining part interacts with the hydrophobic region of the active site (that can accommodate molecules containing 12–13 atoms) (Duplantier et al., 2009). In the hDAAO-inhibitor complex, the side chain of Tyr224 is shifted toward the inner part of the active site and forms a strong π-π stacking interaction, “sandwich,” between its aryl chain and the *re*-side of the isoalloxazine ring of the cofactor. The strongest interaction is observed when the aromatic rings are slightly displaced [i.e., with 3-hydroxyquinolin-2(1*H*)-one] (Figure 4) and not perfectly stacked (i.e., with benzoate). When saturated analogs of these compounds are used, a drop in the binding affinity is apparent, indicating the relevance of the π-π stacking interaction.

The binding of aromatic carboxylic acids to the hDAAO holoenzyme inhibits the flavoenzyme and perturbs its absorbance spectrum in the visible region. For example, benzoate yields a shoulder at ~497nm (K_d_ = 7μM and K_i_ = 9 7μM) (Kawazoe et al., 2006; Molla et al., 2006); anthranilate binding generates a spread classical charge transfer band at ~580nm (K_d_ = 40μM) (Molla et al., 2006). The ligand-binding site is present in the apoprotein form, too, as made apparent by the alteration in protein fluorescence and in thermostability of the latter hDAAO form when the substrate D-serine or the substrate analog tri-fluoro-D-alanine is added (Caldinelli et al., 2009, 2010).

Conversely to benzoate, for the binding of the inhibitor 6-chloro-benzo[d]isoxazol-3-ol (CBIO) (Figure 4) to hDAAO a single-step binding process is evident and the K_d_ value estimated following the quenching of protein fluorescence intensity corresponds well to the K_d_, IC_50_, and K_i_ values determined using different methods.

ADP and CPZ (Figure 4) (IC_50_ of 580 and 5μM, respectively) behave as FAD-competitive inhibitors for binding to hDAAO (Iwana et al., 2008; Sacchi et al., 2008; Terry-Lorenzo et al., 2014). In particular, CPZ binding generates a protein conformation more sensitive to proteolysis and thermal unfolding than the native holoenzyme (Caldinelli et al., 2010). The near-UV CD spectra show that the tertiary structure of hDAAO-CPZ complex differs from that of the hDAAO-FAD: the former more closely resembles that of the apoprotein (Caldinelli et al., 2010).

Notably, the ligands D-serine, FAD, benzoate, and CPZ did not affect the formation of the hDAAO-pLG72 complex (see below).

## Modulation of hDAAO activity

### By protein interaction

Human flavoenzyme function is modulated by interacting with various proteins. hDAAO, through the PTS1-type peroxisomal-targeting signal, interacts with the Pex5p receptor, a protein involved in protein import and in the assembly of peroxisomes (Ghosh and Berg, 2010).

Genome-wide association studies and meta-studies in different populations have linked polymorphisms in the gene encoding pLG72 protein with schizophrenia and other psychiatric diseases (Drews et al., 2013; Sacchi et al., 2016; Pollegioni et al., 2018). In particular, hDAAO specifically binds to the primate-specific protein pLG72: two hDAAO homodimers interact with two pLG72 molecules, yielding a 200-kDa protein complex (K_d_ = 0.08–0.53 μM); for a recent review (see Pollegioni et al., 2018). *In vitro*, the formation of the 200-kDa complex does not alter the kinetic parameters or the binding with the FAD cofactor of hDAAO, but rather induces a change in its overall tertiary structure, causing a time-dependent inactivation (Sacchi et al., 2008). By using low-resolution techniques (i.e., limited proteolysis coupled to mass spectroscopy and cross-linking experiments) structural elements involved in forming the interface surface in the hDAAO-pLG72 complex have been identified, highlighting the role of the N-terminal region of pLG72 in forming the oligomerization interface (Birolo et al., 2016; Sacchi et al., 2017). hDAAO in transiently transfected glial cells (i.e., the U87 human glioblastoma cell line) is largely localized in peroxisomes but also present in cytosol (Sacchi et al., 2011) while pLG72 shows a mitochondrial localization. We proposed that, in this model cell system, newly synthesized hDAAO interacts with pLG72 on the cytosolic side of the outer mitochondrial membrane (Sacchi et al., 2011). Such an interaction increases the D-serine/total serine ratio and decreases hDAAO activity and half-life, see above (Sacchi et al., 2008, 2011; Cappelletti et al., 2014). We recently proposed that pLG72 (itself or recruiting further proteins) might target the cytosolic form of hDAAO to the ubiquitin-proteasome system, thus starting its degradation (Cappelletti et al., 2014). This mechanism could represent a further process to regulate the D-serine levels in the hindbrain where the flavoenzyme is expressed in glial cells.

Analogously, the activity of hDAAO is negatively regulated by bassoon, a component of the cytoskeletal matrix, mainly located at the presynaptic active zone. The hDAAO-bassoon complex formation has been proposed to prevent D-serine depletion acting on the active, extraperoxisomal enzyme form located at presynaptic terminals (Popiolek et al., 2011). The inhibitory effect of bassoon may account for the difficulties in detecting hDAAO activity in the forebrain (Verrall et al., 2007), a region where the enzyme is mostly expressed in neurons.

### By hDAAO inhibitors

Abnormal changes in hDAAO activity yielding locally decreased D-serine levels have been correlated with neurological disorders (e.g., schizophrenia); therefore, the identification of hDAAO inhibitors (to slowing down the neuromodulator degradation process) to be used as drugs has garnered growing interest. This treatment has beneficial effects on cognition and learning functions (Hopkins et al., 2013b).

More than 500 substrate-competitive inhibitors have been identified so far (Gilson et al., 2016). Analogously to the substrate, their chemical structure contains a planar moiety which interacts with the active-site residues close to the FAD cofactor isoalloxazine ring and a second portion which is positioned in the substrate side-chain binding pocket. The “core” of the planar moiety is usually formed by one or two fused rings (one of which might be aromatic) and contains at least a carboxylic group to establish the H-bond interaction with Arg283. The second part of the inhibitor molecule corresponds to the side chain of the substrate: this portion, depending on the size and chemical features, forms further interactions with residues belonging to the substrate specificity pocket and/or to the active site entrance.

A comprehensive review about the details of inhibitor binding to hDAAO has been published recently (Molla, 2017). It ranks classical and novel compounds in four classes:

(i) classical inhibitors: typically single-ring ligands, i.e., benzoate, anthranilate, and improved variations (Sacchi et al., 2012; Katane et al., 2013). The compound 5-methylpyrazole-3-carboxylic (Figure 4) acid is a prototype of this class of compounds in which two C atoms of the ring are substituted by N: the optimized H-bonds network allows a high affinity for hDAAO (K_i_ = 0.39 μM). This compound crosses the blood-brain barrier in rats, thus raising the D-serine level in certain brain regions (Adage et al., 2008);
(ii) second-generation inhibitors: larger compounds than classical inhibitors since they are characterized by two substituted, heterocyclically fused rings, which form additional H-bonds and van der Waals interactions with residues forming the active site, i.e., compounds derived from 3-hydroxyquinolin-2(1H)-one and CBIO (Ferraris et al., 2008; Katane et al., 2013). The main drawback of the last compound is the low passage through the blood-brain barrier: such a compound does not increase D-serine levels in the brain (Ferraris et al., 2008);
(iii) third-generation inhibitors: bulky and flexible compounds whose side chain binds to an additional “subpocket” at the entrance of the active site generated by a conformational change in Tyr224 induced by ligand binding (Raje et al., 2013; Terry-Lorenzo et al., 2014);
(iv) novel-generation inhibitors: molecules that can interact with the hDAAO-pLG72 complex since they contain the “ebsulfur” (2-phenyl-2,3-dihydro-1,2-benzothiazol-3-one) substructure that forms S-S thiol bonds with the cysteines of hDAAO, when the protein is partially unfolded due to pLG72 binding (Terry-Lorenzo et al., 2015). Compound [2-(2,5-dimethylphenyl)-6-fluorobenzo[d]isothiazol-3(2H)-on (Figure 4) inhibits hDAAO, acting as both FAD- and D-serine-competitive inhibitor. The so-called “compound 22,” classified as a class C compound by Terry-Lorenzo et al. (2015), only acts as hDAAO inhibitor under oxidizing conditions. This compound does not dissociate from the flavoenzyme in jump-dilution experiments and stably inactivate the enzyme: recovery of the DAAO activity is obtained only by adding a reducing agent.

### By single point substitutions

Based on biochemical properties, hDAAO variants corresponding to known single nucleotide polymorphisms or sequence conflicts have been grouped into two classes: hypoactive and hyperactive; for a recent review (see Sacchi et al., 2018). The conditions and levels of recombinant expression of seven variants of hDAAO are reported in Table 2 of Sacchi et al. (2018).

**Figure 5:**
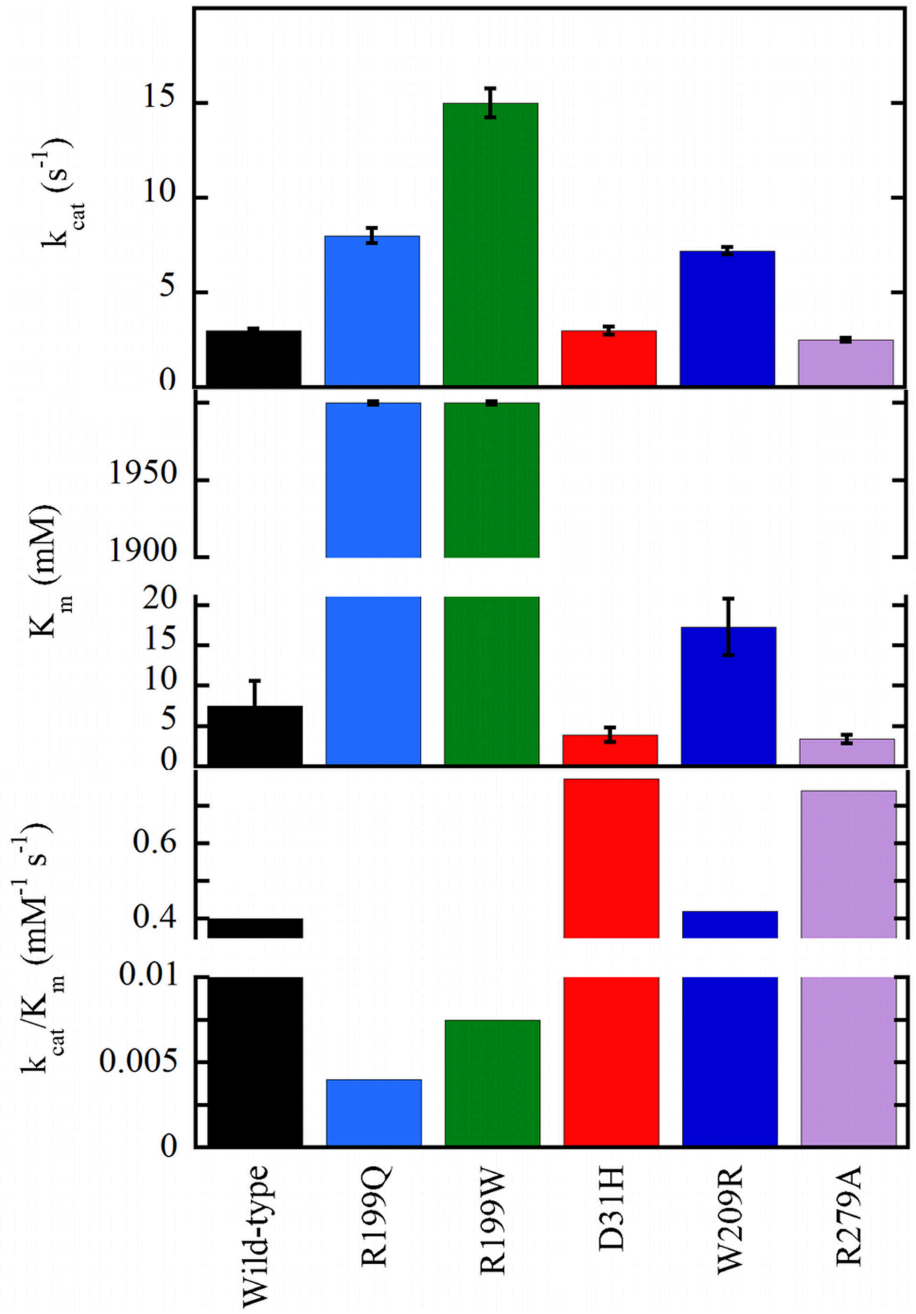
Apparent kinetic parameters of wild-type and hDAAO variants, determined at air saturation (0.25 mM oxygen), 25°C, and pH 8.5 (Molla et al., 2006; Caldinelli et al., 2013; Cappelletti et al., 2015).

The G183R, R199W, and R199Q hDAAOs show significantly decreased enzymatic activity (or fully abolished for the latter variant, see Figure 5), and a perturbation of the conformation: (a) in G183R hDAAO, corresponding to the coding mutation occurring in the ddY/DAAO^−/−^ mice strain expressing the inactive G181R DAAO (Konno and Yasumura, 1983), alterations in secondary structure elements likely alter the conformation of the flavin binding domain and thus negatively affect the cofactor binding (Murtas et al., 2017a); (b) the tertiary structure of R199W (the variant associated with the onset of fALS) and R199Q variants is significantly altered: this favors aggregation propensity but does not modify the interaction with pLG72 (Cappelletti et al., 2015; Murtas et al., 2017a); (c) in G331V hDAAO the change in the C-terminal α-helix promotes protein aggregation, strongly affecting the variant solubility (Caldinelli et al., 2013). At the cellular level, both G183R and G331V variants were partly mistargeted: they formed cytosolic protein aggregates, which largely colocalized with ubiquitin, and resulted in increased apoptosis (Caldinelli et al., 2013; Murtas et al., 2017a).

On the other hand, the D31H, W209R, and R279A substitutions have the opposite effect on hDAAO activity, resulting in slightly or significantly improved catalytic efficiency (Figure 5) and FAD affinity. For example, a 2-fold increased turnover number was apparent for the W209R hDAAO, which was more active than the wild-type hDAAO using 0.3mM D-serine and 5μM FAD, i.e., concentrations resembling physiological conditions (Cappelletti et al., 2015).

**Figure 6:**
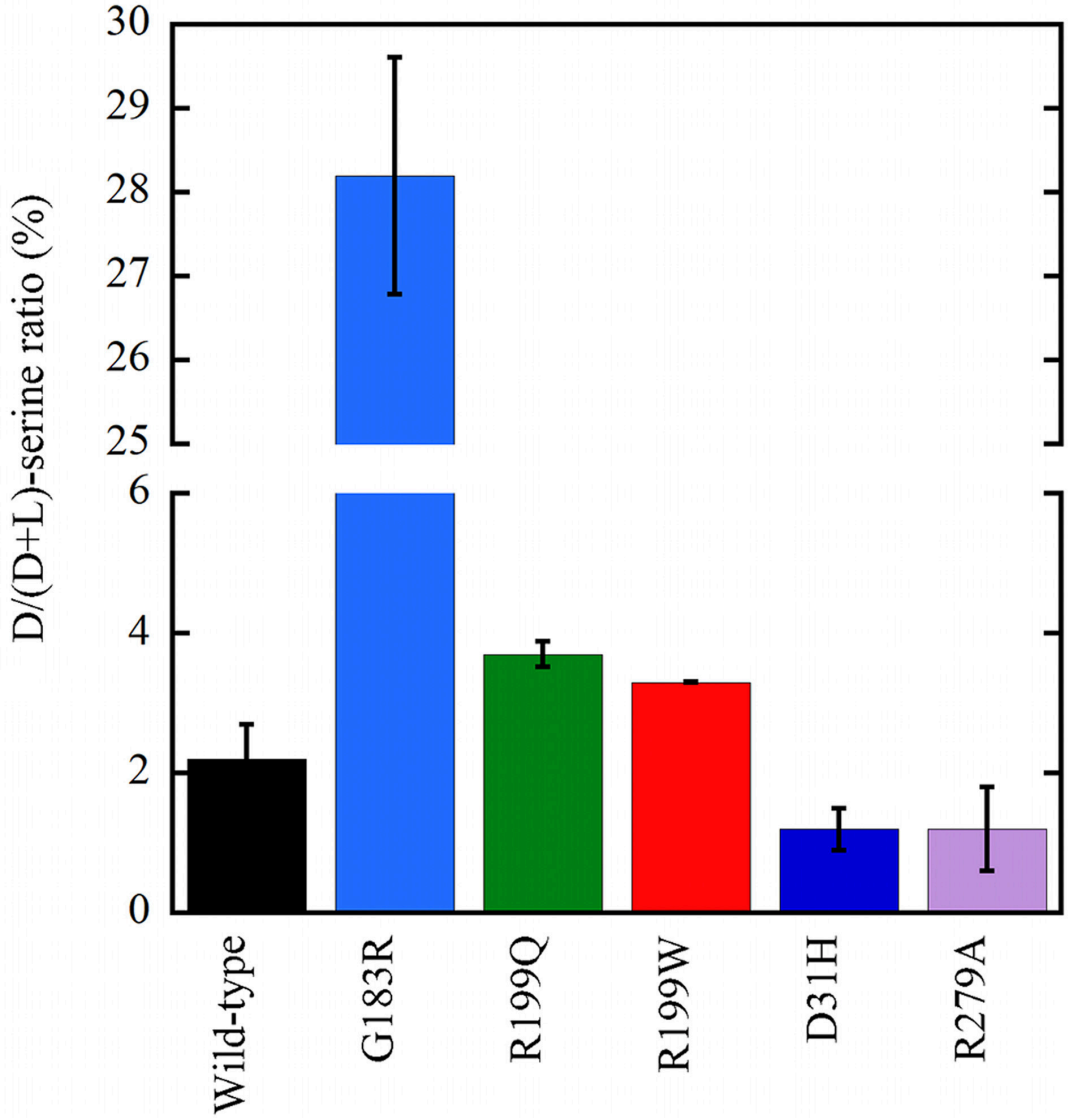
Effect on the cellular D/(D+L) serine ratio in U87 human glioblastoma cells stably expressing EYFP-hDAAO variants (Caldinelli et al., 2013; Cappelletti et al., 2015; Murtas et al., 2017b). The effect of W209R substitution was established on transiently transfected cells (not shown): a figure of 4.2 ± 0.1 vs. 5.8 ± 0.4 was determined at 24 h for the variant and wild-type hDAAO, respectively.

Following overexpression in U87 cells, all the investigated hDAAO variants significantly altered the cellular levels of D-serine (Figure 6; Caldinelli et al., 2013; Cappelletti et al., 2015; Murtas et al., 2017a). The expression of inactive variants of hDAAO could produce susceptibility to neurodegenerative disorders due to augmented D-serine levels which, when paralleled by elevated glutamate levels, could lead to hyperactivation of NMDAR and thus to excitotoxicity. In contrast, a deficit in NMDAR-mediated transmission might be related to the expression of hyperactive variants due to an abnormal decrease in D-serine at the synapses, as proposed in schizophrenia onset. Furthermore, hDAAO hyperactive variants produce nonphysiological levels of H_2_O_2_: this process could contribute to the molecular mechanism of the central sensitization typical of chronic pain.

To delve into the structure-function relationships in mammalian DAAOs, alanine-scanning analysis of first and second shell residues of the enzyme from pig prompted the focus on active-site lid residues (region 221–225) and on the positions 55 and 56 in hDAAO (Subramanian et al., 2018). Molecular dynamics simulations identified a narrow tunnel that could provide access to the active site of hDAAO, named tunnel T1. The Y55 residue was suggested to be involved in anchoring the lid loop in the closed conformation (its dynamics are hampered by Y314), modulating the solvent access and substrate/product exchange at the active site and separating T1 from an additional, putative tunnel. The Y55A substitution facilitated accessibility of the active site: a 2-fold increase in specific activity on D-Trp was observed.

## By post-translation modifications

The molecular mechanisms by which hDAAO expression and acquisition of catalytic activity are achieved inside the cell are still largely unknown: a fine and careful regulation through post-translational modification(s) is expected. Actually, hDAAO was proposed to be regulated by nitrosylation (Shoji et al., 2006). In detail, the activity of DAAO, in a membrane fraction of U87 glioblastoma cells, was enhanced by NO in a dose-dependent manner. The authors proposed that, in astrocytes, NO may inhibit SR and enhance hDAAO activities thus accelerating D-serine degradation. Following D-serine supply from astrocytes to neurons, synthesis of nitric oxide in neurons may temporarily be increased, yielding a feedback regulation of the neuromodulator.

## Conclusions

With the final aim to use hDAAO in different tissues responding to several needs, evolution adopted complicated regulatory strategies to modulate the activity of the flavoenzyme. In human brain tissues, hDAAO should be mainly present in the apoprotein, inactive form considering the physiological concentration of FAD and its weak interaction with the apoprotein moiety. Conversion of the inactive hDAAO apoprotein into the active holoenzyme is facilitated by the presence of an active-site ligand, such as the substrate: this represents an efficient way to maintain the level of selected D-amino acids in the physiological range.

We are conscious that, despite the important role played by hDAAO in main physiological processes, the modulation of its functional properties is still largely unknown. A main issue is the modulation of the activity by post-translational modifications (as known for serine racemase) and by further interacting proteins. A second matter is the role of hDAAO activity in important human diseases. Here, a way to elucidate links with cell functions is represented by the investigation of the role of epigenetic modifications on *DAO* gene expression in different cells and tissues during development and pathological conditions. In this regard, a CpG methylation analysis of the *DAO* promoter was performed recently and brain region-specific epiallelic profiles were detected in schizophrenic patients and healthy controls (Keller et al., 2018). These different methylation signatures have been proposed to be indicative of cell populations containing the *DAO* gene in different functional states.

The known properties of hDAAO strengthen our belief that the flavoenzyme activity must be finely tuned to fulfill a main physiological function such as the control of D-serine levels in the brain.

## Author contributions

LP designed the review. All authors analyzed the literature and wrote the manuscript.

### Conflict of interest statement

The authors declare that the research was conducted in the absence of any commercial or financial relationships that could be construed as a potential conflict of interest.

## Abbreviations

ALS: amyotrophic lateral sclerosis
CBIO: 5-chloro-benzo[d]isoxazol-3-ol
fALS: familial amyotrophic lateral sclerosis
DAAO: D-amino acid oxidase
hDAAO: human DAAO
NMDAR: N-methyl-D-aspartate type glutamate receptor
SR: serine racemase.
